# Research on Control Method of Waste Heat Utilization System Based on Multi-parameter Coupling

**DOI:** 10.1038/s41598-022-15808-0

**Published:** 2022-07-07

**Authors:** Yanjun Xiao, Kun Zhang, Yameng Zhang, Wei Zhou, Weiling Liu, Feng Wan

**Affiliations:** School of Mechanical Engineering, Hebei University of Technology, Tianjin, 300132 China

**Keywords:** Energy science and technology, Energy harvesting, Energy infrastructure, Computer science, Software

## Abstract

The recovery of low-quality waste heat is a major problem in energy utilization. In order to solve this problem and improve energy utilization, the research group designed a low-quality waste heat power generation device with Roots power machine as the core. However, the device has poor ability to adjust the rotation speed and it is difficult to generate electricity stably. The fundamental reason is that the system has many variables and strong coupling. According to the actual working conditions, the power of the device is 10 kW, and the fluctuation range should be within ± 7%. On the one hand, it can be improved by hardware, on the other hand, the design of software is also very critical. At present, through the investigation of domestic and foreign researches on the control system, it is found that the stability of the system is gradually improved, but the problem of strong coupling between variables has not been effectively solved. Therefore, the research group modeled the variables in the system and obtained a coupled model. Based on the couple model, the research group introduced nonlinear multi-model adaptive closed-loop decoupling control and designed a control system. The simulation results show that the maximum overshoot of the control system is 3.9%, the adjustment time is also reduced, and it is stable in low quality waste heat recovery device. Experimental results show that under the control of the system, the rotational speed of roots motor can keep stable, the maximum deviation is not more than 21.4 r/min, and the fluctuation range is within ± 7%, which meets the requirements of the index. This has laid the foundation for the follow-up research of grid-connected power generation.

## Introduction

### Research background

With the rapid development of society, people's demand for energy is also increasing. At present, the global use of clean energy accounts for less than 18%, and the large-scale use of primary energy, especially fossil energy, is still the main energy lifeline of current industrial development. In the process of using fossil energy, on the one hand, it will cause pollution and damage to the environment. On the other hand, due to efficiency issues, a large part of the energy will be lost to the surrounding environment in the form of heat. Among the lost waste heat, part of it is easier to recover due to its higher temperature. Now many industries already have industrialized recovery methods, such as sinter waste heat recovery technology in the steel industry and steel slag waste heat recovery technology Etc.; screw expansion power machine power generation technology in the coking industry; low-temperature waste heat recovery power generation technology in the cement industry, etc. However, for the recovery and utilization of low-quality waste heat with a temperature not exceeding 160℃ and a pressure not exceeding 0.8 MPa, the above methods are difficult to effectively recover it, so a considerable part of the low-quality waste heat is wasted.

**Figure 1:**
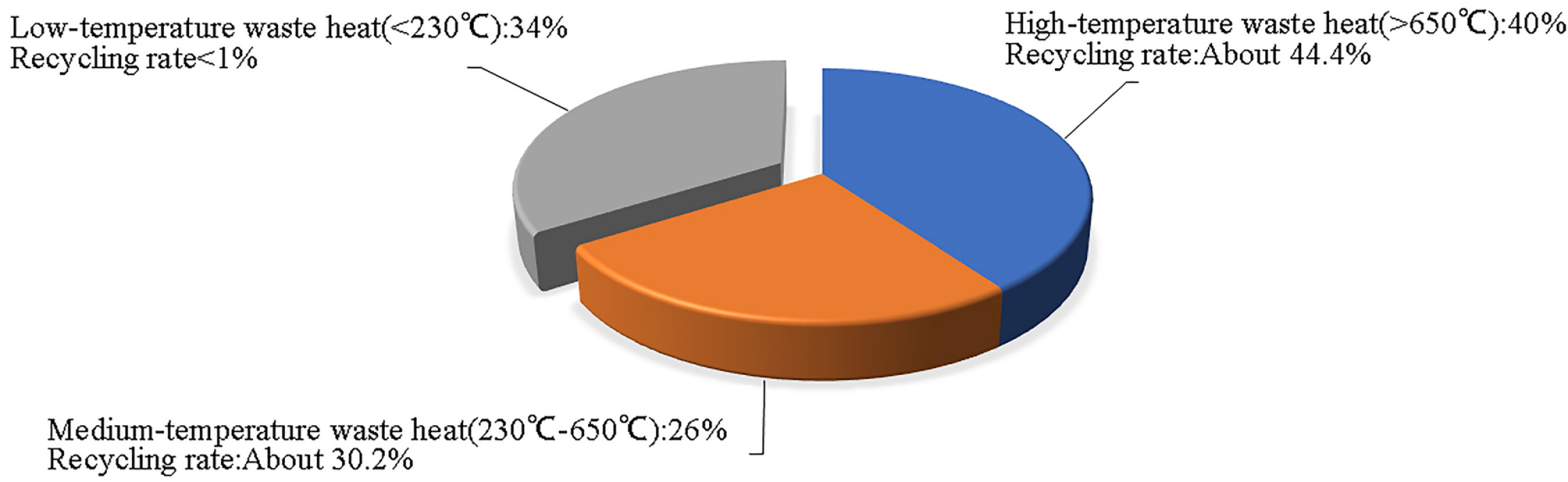
Distribution and reuse of waste heat resources.

Industrial waste heat resources are widely distributed in many industries such as iron and steel, metallurgy, building materials, non-ferrous metals, petrochemicals, light industry, etc. It is currently a recyclable resource with the most widespread distribution and the greatest application potential in industrial production. Industrial waste heat is a kind of secondary energy. It is the heat lost in the industrial production process of primary energy. It is generally discharged into the external environment in the form of flue gas, waste gas, and wastewater^1^. According to statistics, the total amount of waste heat resources in the metallurgy, building materials and chemical industries is relatively large, reaching about 80%; medium and low-quality waste heat resources account for about 54%, and the annual utilization rate is about 2.7 million tons of standard coal^2^. As shown in Fig. 1, high-, medium-, and low-temperature waste heat accounted for 40%, 26%, and 34%, respectively, but their secondary utilization rates are quite different. Among them, the medium and low temperature waste heat is widely distributed, but due to its low quality, the recovery rate is much lower than the high temperature waste heat, which limits the further improvement of the overall utilization rate of industrial waste heat^3^. Research on low-quality waste heat recovery technology is conducive to comprehensive conservation and efficient use of resources, promote the development of low-carbon cycles, advance the energy revolution, accelerate energy technology innovation, and build a clean, low-carbon, safe and efficient modern energy system. Energy saving, emission reduction and environmental protection will be an important part of economic development in the future.

### Literature review

At present, the research on low-quality waste heat recovery in universities and scientific research institutions mostly uses screw expanders and scroll expanders as core equipment, and most of the research on waste heat recovery is the improvement and optimization of existing solutions. However, these studies have obvious shortcomings when applied to low-quality waste heat recovery, which are mainly manifested in the complex structure of the core equipment, high processing costs, inconvenient maintenance, and high operating costs. As a result, these technologies and equipment are not widely used in low- and medium-quality waste heat utilization systems, and they cannot meet the needs of small and medium-sized enterprises for energy-saving technologies^4^. In addition, while the mechanical structure of waste heat recovery device is studied abroad, it is also gradually studied in the micro direction. On the one hand, the heat transfer effect can be improved by adding nano-particles or nano-fluids; on the other hand, the heat transfer efficiency can be improved by improving the radiator at the nano-level. Ibrahim Muhammad studied stretchable rotating discs with heat transfer functions and carried out numerical analysis of their fluids^5,6^. Zhixiong Chen et al. tested 27 refrigerants and studied a thermal conductivity model with better accuracy^7^. Subsequently, nano-particle fluids such as copper oxide or alumina were added into the heat transfer system, and thermodynamics laws and exergy were analyzed. The analysis results show that adding nanoparticles into the heat exchanger fluid can reduce exergy loss and reduce the efficiency of the second law of thermodynamics, so as to improve energy conversion efficiency^8–13^. This kind of method also plays a positive role in waste heat utilization technology, but also has the disadvantage of high cost.

**Figure 2:**
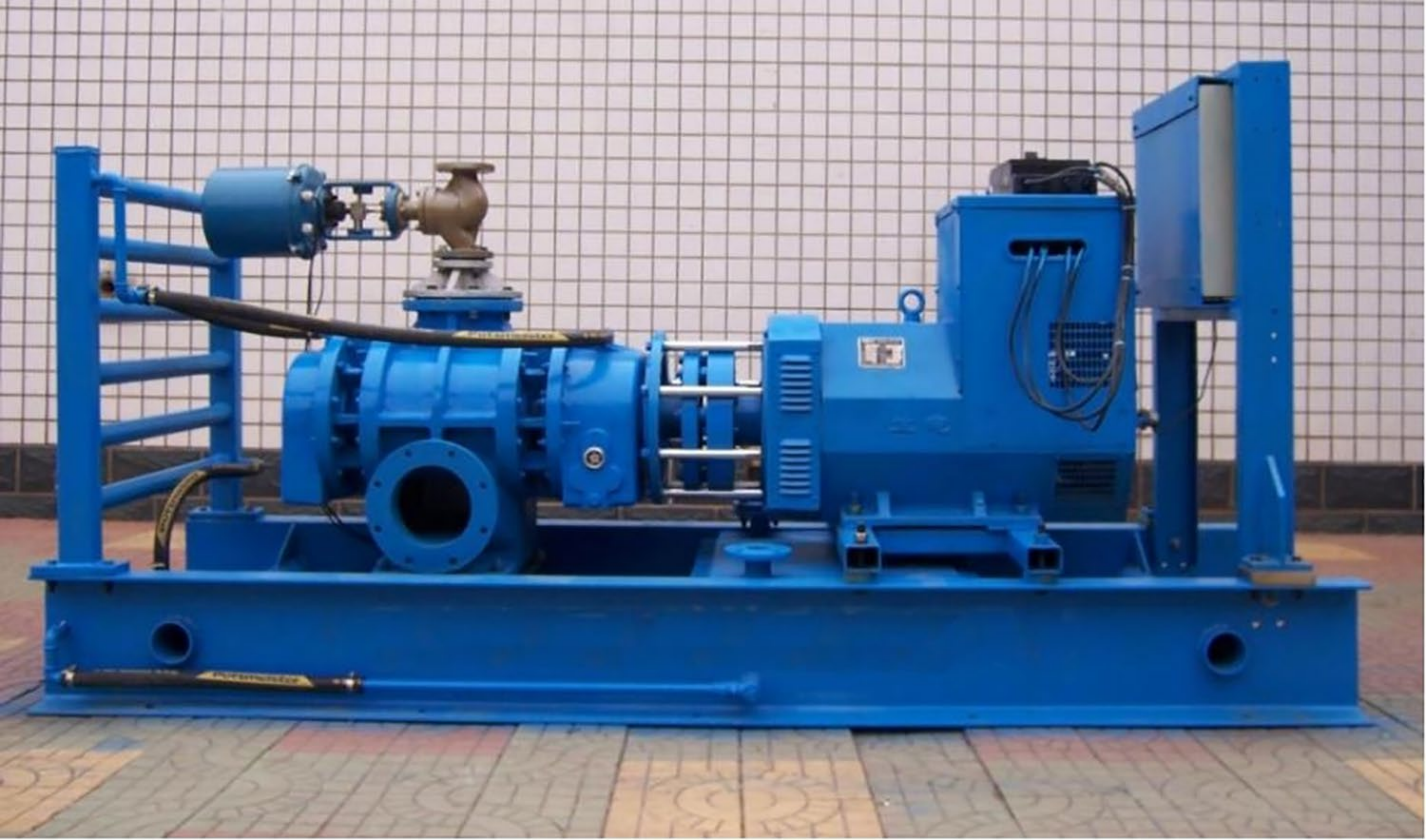
Low-quality waste heat recovery and utilization device with roots power machine as the core.

In response to the technical requirements of low-quality waste heat recovery and utilization, the research group has developed a new type of Roots-type power machine and used it as the core equipment for waste heat recovery and utilization.It has been verified through experiments that it can be used for the recovery and utilization of low-quality waste heat. The device is shown in Fig. 2. At present, the waste heat recovery process of the device and its control system have been studied, but the existing research is not deep enough. Although the existing control method can solve the problem of the operation of the waste heat recovery device, it is difficult for the existing control method to return the system to the preset rated state at a faster speed and a smaller overshoot when the air source fluctuates. The fluctuation of the gas source will cause the output power to fluctuate. If this fluctuation is not controlled, it will cause the waste heat recovery device to become overloaded during energy conversion. In most cases, the recovered waste heat will be used for power generation, and when the waste heat recovery device is overloaded, the connected electrical equipment is bound to be affected. Therefore, this subject intends to study a control method to solve the problem that the low-quality waste heat recovery device cannot work stably when it is disturbed.

The low-quality waste heat has small scale, frequent fluctuations, low specific heat capacity, and the fluctuation range is more severe than that of medium and high temperature waste heat, which makes it difficult to stabilize the operating state of the Roots waste heat power generation device. Irregularly fluctuating air source, the large inertia of the Roots power machine, the strong coupling effect of temperature and pressure and other parameters, coupled with the different time and location of the external environment, will cause the Roots waste heat power generation device to produce irregular deviations in the output power. When the deviation is severe, it may even cause partial load or generation trip. In order to make the Roots power machine run stably, it is necessary to decouple the variables that affect the rotation speed of the Roots power machine. With the decoupling model, the control effect of the control system will be more accurate and stable.

Traditional decoupling methods are mainly suitable for linear time-invariant multivariable systems. The basic idea of designing decoupling method is to build a decoupling network, and make the transfer function matrix between input and output variables become diagonal matrix, so that the system is easier to control. Adaptive decoupling control strategy is a combination of adaptive control technology and decoupling control technology, that is, the decoupling, control and identification of the controlled object is combined to achieve precise decoupling control of the system with unknown or time-varying variables. In essence, the coupling term can be regarded as measurable interference, and the coupling action, static compensation and compensator parameters can be optimized by self-correcting feedforward control method. Adaptive decoupling has been applied in many engineering fields, but its application scope is limited because of the need for online identification of target model, complex algorithm, large amount of calculation, poor adaptability to dynamic modeling and process disturbance, and weak robustness of the system^14^.

In the aspect of control system, the control system of low quality waste heat recovery and utilization system is mainly embedded control system. The embedded controller has many advantages such as small volume, high reliability, powerful function and easy to use. Zhang Wen et al. studied the dynamic performance of internal combustion engine—organic Rankine cycle combined system in the waste heat recovery system of internal combustion engine. The closed-loop proportional integration and feedforward control are adopted. The response time and overshoot of PI control are estimated and compared with that of feedforward control alone. The results based on the World Coordinated Transient cycle (WHTC) show that the designed closed-loop PI control has shorter response time and better tracking ability in the dynamic process^15^. Pang Kuo Cheng et al. constructed a 3 kW organic Rankine cycle test rig engineering simulator based on the experimental data of R245fa, R123 and their mixtures. The simulation performance of pump and expander is verified by experimental results, and the influence of mass flow rate is discussed. The results show that the proposed overheat control strategy can obtain the best operating conditions. Frequency conversion control strategy is preferred for small ORCS. It indicates that the organic Rankine cycle engineering simulator is a good tool to predict the operation characteristics of the organic Rankine cycle, and can further guide the advanced evaluation and long-term variation^16^. In the Rankine cycle technique, Toffolo proposed a hybrid evolution/traditional optimization algorithm, which considered the heat transfer constraints in the pipeline. Using his algorithm, a waste heat recovery system model with good tracking ability can be obtained^17^. Quoilin and Lemort et al. have modeled the organic Rankine cycle based on a vortex expander. Through this model, they confirmed that the organic Rankine cycle is particularly suitable for recovering low temperature waste heat, and also pointed out through experimental analysis that the main losses affecting the performance of expander are internal leakage, supply pressure drop to a lesser extent, and mechanical losses^18–20^. Jaume Fito, Sacha Hodencq et al. found that the waste heat temperature was correlated with the capacity of the heat storage device and optimized it to improve the waste heat recovery rate^21^. In the waste heat recovery system of power plant, the designer uses PLC as the controller and WinCC configuration software as the upper computer to develop the waste heat recovery monitoring system. In addition to controlling waste heat recovery, monitoring parameters can also be displayed in real time on the screen of the upper computer^22^. Designers optimize the traditional PID control method and develop a waste heat recovery control system based on fuzzy PID control strategy. More accurate control of parameters is achieved, and the energy consumption of the control system is also reduced^23^. In the diesel engine waste heat recovery system, the researchers used the MotoTron rapid prototyping development platform to better control the exhaust through an optimized PI closed-loop control strategy to achieve fuel savings. In addition, fault diagnosis and alarm functions are added to the system to monitor possible abnormal situations^24^. Zhao Mingru proposed a set of map-based feedback closed-loop control algorithm for the waste heat recovery system of internal combustion engine under driving conditions. Firstly, the model order reduction method was adopted to simplify the initial organic Rankine cycle model into a reduced order model that can be used for control without losing excessive accuracy. Then, the rolling time domain optimization is combined with the particle swarm optimization algorithm to form the nonlinear model predictive controller. Finally, the nonlinear state estimator constitutes the final feedback link, and the control effect is greatly improved^25^.

According to the summary above and the research on the control system of the existing waste heat recovery device, the current control strategy is mainly divided into the following aspects:

(1) PID control strategy.Now PID control strategy is the most widely used control method in the actual industrial production process. In the application of PID control strategy, the effect of PID control depends largely on the parameters of PID controller. In addition, PID control involves few controlled parameters and the signal processing process is relatively simple. Therefore, combined with the previous summary, most scholars adopt PID or improved PID control method in preliminary study of waste heat recovery control system^26^.

However, PID control method considers too few factors in the signal processing process, and there is no good method to generate differential signal. In traditional PID control strategy, the function of error integral feedback is to eliminate static error, so as to improve the accuracy of system response. At the same time, the closed-loop system becomes insensitive due to the introduction of system error integral feedback. When the traditional PID control method is applied to the low-quality waste heat recovery system, the system is prone to oscillations, which eventually leads to pulsating air flow in the pipeline^27^.

(2) Optimized PID control strategy. Due to the defects of traditional PID control strategy, the research on PID optimization is very rich. For example, the closed-loop PI control and fuzzy PID control mentioned above are optimized on the basis of PID control. The control signal of the traditional PID control strategy is directly obtained by the difference between the set value and the output feedback value, which leads to the contradiction between the rapidity of response and the overshoot. Active disturbance rejection technology is derived from the process of PID optimization^28^.

For the waste heat recovery power generation system, there are many variables in the system, which is a multi-input and multi-output system, and there are many variables associated with each other, with strong coupling. The optimized PID controller usually does not need an accurate mathematical model, but the coupling between different variables will lead to the reduction of the robustness of the controller in the adjustment process^29^.

(3) Decoupling control strategy. As the control system becomes more and more complex, the variables in the control system also become more and more, and the coupling between the variables in the control system becomes more and more prominent. Variable coupling is a common phenomenon in industrial control system. Because of the coupling between variables, it will not only increase the difficulty of industrial system control, but also greatly reduce the control effect of the system, and even lead to the collapse of the whole system in serious cases. Therefore, decoupling strategy in the control system has become one of the important means to improve the controller performance and meet the requirements of the control process.

Traditional decoupling methods are mainly suitable for linear time—invariant multivariable systems. The basic idea of variable decoupling design method is to construct a decoupling network, calculate the transfer function of multi-input and multi-output control system, and make its transfer function matrix into diagonal matrix, reduce the complexity of control system design.

Adaptive decoupling control strategy is a new control strategy derived from the integration of adaptive control technology and decoupling control technology, which combines the coupling analysis, control and identification of the controlled object to achieve more accurate control of the system containing unknown variables or time-varying systems. The essence of adaptive decoupling control technology is that coupling variables are regarded as measurable interference variables, and the coupling actions, static compensation and compensator parameters of the control system are optimized by the feedforward control method with self-correction function. There are many typical application examples of adaptive decoupling control in the field of engineering, but due to the need for online identification of target model, complex algorithm, large amount of calculation, poor adaptability to dynamic modeling and process disturbance and weak system robustness, the application scope is limited to a certain extent^30^.

(4) Intelligent algorithm fusion control strategy. In other words, control system design is carried out through artificial intelligence control algorithms, including pinch point technology, nonlinear programming, multi-integer linear programming, genetic algorithm, artificial neural network, multigeneration system and many other different methods^31^. Using self-learning artificial intelligence control algorithm can achieve more intelligent control effect, but with the gradual improvement of control effect, the complexity of control system design is getting higher and higher.

In addition, intelligent algorithms also have their own advantages and disadvantages. Therefore, the intelligent algorithm fusion control strategy utilizing the complementary advantages of different algorithms is gradually developed. In the study of waste heat recovery, Xiao Yanjun et al. found that Long Short-Term Memory(LSTM) often had high accuracy and sensitivity in the aspects of pre-processing, feature selection and data analysis, and had a strong ability to deal with strongly coupled data. Long and short term memory model can deal with a large amount of data effectively and improve the response speed of control system while predicting data. When internal model control is applied to large time delay systems, the control effect has obvious advantages compared with other control strategies, and has good tracking performance and anti-interference ability. Therefore, an algorithm fusion control strategy based on deep learning and internal model control is proposed^32^. Compared with the single control algorithm, the stability of the algorithm fusion control strategy has been significantly improved. Similarly, the complexity and development cycle of the system are several times higher than before.

To sum up, in order to improve the heat recovery efficiency of the waste heat recovery device and optimize the stability of the system, on the one hand, it is necessary to decoupled multiple variables with strong coupling in the system and analyze the relationship between different variables. On the other hand, combining the decoupling algorithm with the control algorithm to control the waste heat recovery system on the basis of decoupling can effectively improve control accuracy and stability.

## Research gap and motivation

In summary, There are two gaps in the field of low-quality waste heat recovery, one is the gap in the equipment of low-quality waste heat recovery, the other is the gap in the control system. Through self-development, the research group designed Roots power machine for low-quality waste heat recovery, which solved the problem of waste heat recovery equipment to a certain extent. On the device, however, problems with the control system have not been resolved. One of the main problems is the strong coupling of variables in the system. Therefore, this paper aims to study a decoupling based control method that can control the stable operation of waste heat recovery equipment.

The research group have made a certain summary of the characteristics of the control system through research and literature review. The common control method has some defects in the stability, and the optimized control method can improve the stability of the control system to a certain extent. In contrast, the control strategy based on algorithm fusion can effectively improve the stability of waste heat recovery, in which variable decoupling is an important step. For low quality waste heat recovery power generation device, the stability is one of its important indicators, so the algorithm fusion method based on decoupling control can enhance its stability.

### Contribution and paper organization

The low quality waste heat recovery device studied in this paper still has problems in the stability of energy output. Therefore, the main purpose of this paper is to enhance its stability by improving the control system. The low stability of the system is closely related to the strong coupling of variables in the system. Therefore, this paper proposes a closed-loop decoupling control strategy based on multivariable coupling. The experimental results show that this method can effectively improve the stability and tracking performance of waste heat recovery system. On the one hand, this achievement is conducive to the realization of power supply for small equipment, on the other hand, it also provides the possibility for the future grid power generation.

The first chapter of this paper describes the research background and status quo of the subject, summarizes the research blank, and puts forward the research content, purpose and significance of the subject. The second chapter introduces the structure, working principle and process of Roots type waste heat recovery system, and shows the working characteristics of waste heat recovery system. The third chapter is the analysis of system variables and coupling model. Chapter 4 is the design of nonlinear multi-model adaptive closed-loop decoupling control system based on coupling model. The fifth chapter is the simulation and experimental research, through the simulation and experimental verification, the advantages and disadvantages of the new control strategy in the control performance. The last part is the summary of this topic.

## Structure and index of roots waste heat power generation device

**Figure 3:**
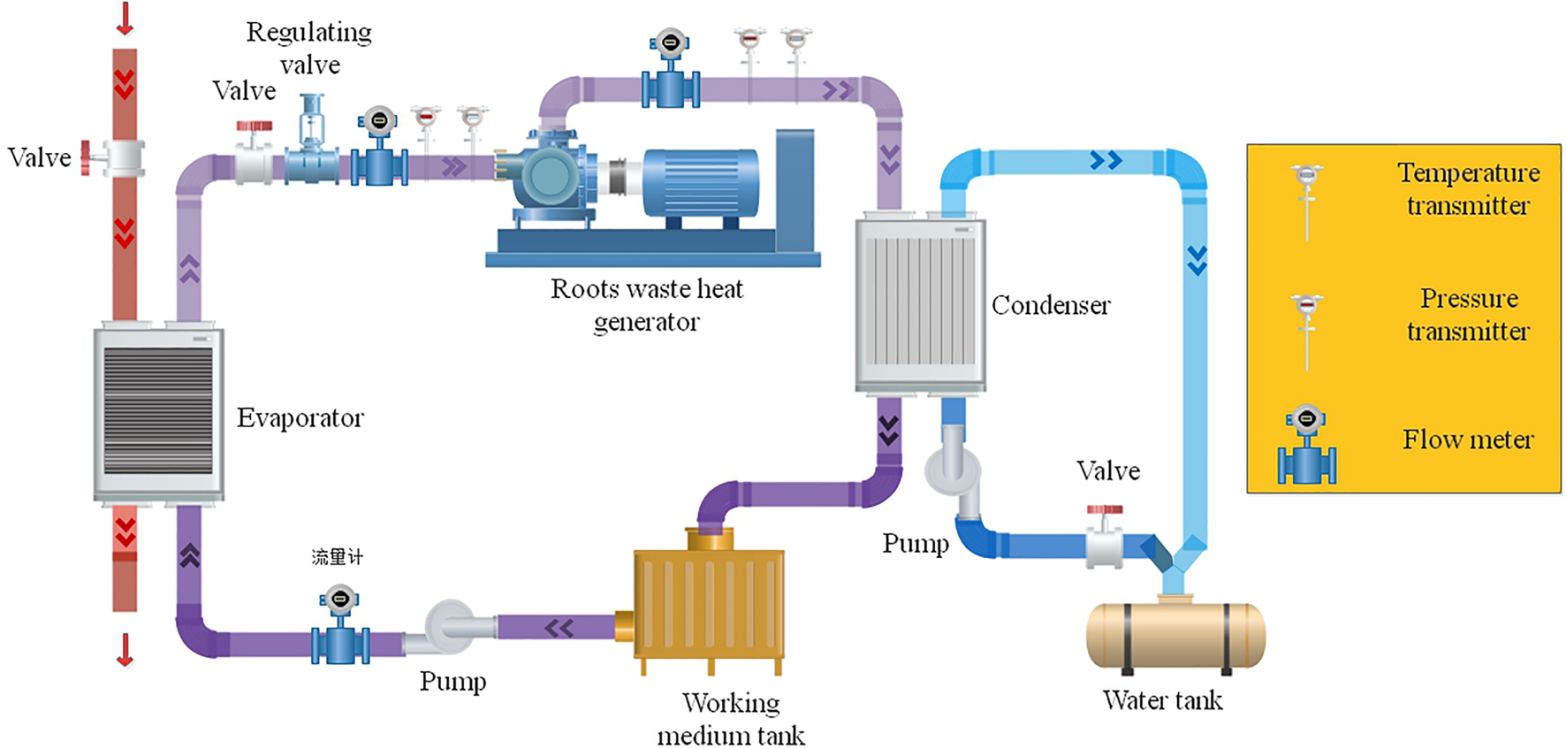
Roots waste heat recovery power generation device.

The Roots waste heat power generation device is a power generation device that uses a Roots power machine as the power mechanism, and relies on the expansion of the working medium to drive the Roots power machine to rotate to realize the conversion of air source energy to mechanical energy. The research object of this paper is Roots waste heat power generation device independently developed by the research group, as shown in Fig. 3.

The device is mainly composed of Roots power machine, generator, regulating valve, sensor, transmission pipeline and connecting parts and other equipment. Its working principle: the gas source first enters the evaporator to exchange heat. The working fluid after heat exchange enters the Roots power machine after being adjusted by the regulating valve. The blades of the Roots power machine are driven by the expansion of the working fluid gas and the pressure difference, and the connecting parts such as the coupling drive the generator shaft to rotate to generate electricity. Its working condition parameters are shown in Table 1.

**Table 1:** Operating condition parameters of Roots waste heat power generation device.

Parameter index	Numerical range
Rated power (kW)	10
Intake pressure (MPa)	0.2–1.0
Air source temperature (℃)	80–160
Output allowable fluctuation	± 7%

The working state of Roots waste heat power generation system is restricted by the fluctuating state of working fluid gas. In addition, the physical and chemical processes involved in the work of the Roots power machine are closely related and complex. To ensure the stability of the rotation speed of the Roots power machine and the safe and efficient operation of the power system, it is necessary to keep all the parameters of the system working in coordination and at the required level. The device is suitable for generators with rated power below 100 kW (according to the gas flow per unit time), and the rated power of the experimental device selected in this paper is 10 kW.

## Parameter analysis of system control

### Analysis of system variables

The output speed of the Roots power machine is directly related to the output of the system's electrical energy, so the key to the quality of waste heat power generation lies in the stable control of the output shaft speed of the Roots power machine. This output is affected by factors such as the temperature, pressure, and flow rate of the working fluid, the temperature and pressure of the gas outlet, and the slagging, ash, and fouling conditions on the inner cavity of the power machine.

**Figure 4:**
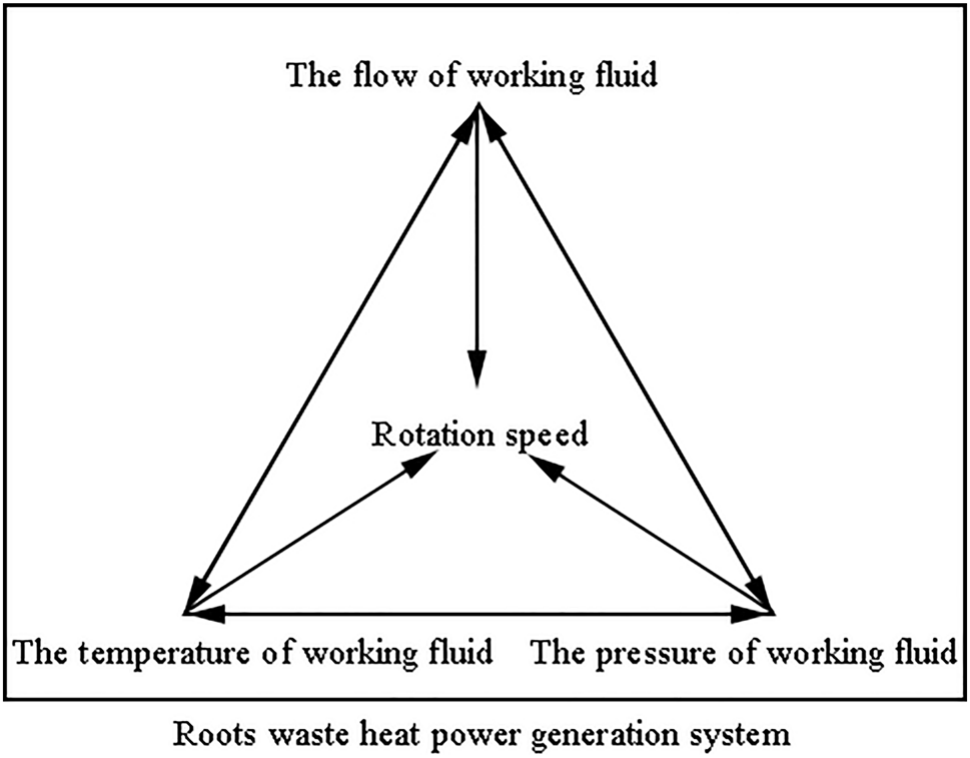
Variable coupling diagram of Roots waste heat power generation system.

The Roots waste heat power generation system is a three-input one-output system, and the coupling correlation of the input and output variables of the system is shown in Fig. 4. The valve opening will affect the working fluid flow and the inlet pressure, working fluid temperature, working fluid flow and working fluid pressure are related to each other, which together with the exhaust air flow rate affect the rotation speed of Roots power machine. Due to the serious coupling of parameters in the work process of the Roots power machine, the adjustment of the system operating variables involves a chain reaction of changes in multiple parameters, making it more difficult to control the controlled variables.

Specifically, in the Roots waste heat power generation process, the effects caused by fluctuations of certain parameters are consistent. For example, an increase in temperature will cause an increase in the pressure in the intake duct of a Roots power machine, and under the same conditions, it will cause an increase in the work done by the power machine. These are basic effects, and the signal changes are basically the same. Thinking at this level can confirm that the changing laws of the system are closely related to the exact working points and working parameters of each device.

In the process of doing work, the working fluid gas flow rate is fast, and the working conditions change rapidly. In contrast, the adjustment effect of the control equipment has obvious hysteresis. On the one hand, when the flow of the working fluid in the device is adjusted, the internal pressure will reversely change in a short time, so that the flow change of the working fluid inside the device has a certain lag compared with the expected. On the other hand, the control mechanism of the electric control valve makes the system have pure lag, which will not only make the system control not timely, increase the dynamic deviation, but also endanger the stability of the system.

### Variable coupling analysis based on control variable method

In order to better explore the work characteristics of Roots power machines, it is necessary to carry out model analysis on the physical quantities and coupling relations involved in the work process. The relationship between generator power and speed can be expressed as:

3.1 $$P = \frac{n}{9550}M$$

In the formula, *P* is the rated power of the generator, *n* is the rated speed, and *M* is the rated torque. According to formula 3.1, in an ideal state, the relationship between the power of the generator and the rotation speed can be considered as a linear relationship, that is, the relationship between the rotation speed of the Roots power machine and the power of the generator is also a linear relationship.

In order to further explore the relationship between the working fluid flow rate, working fluid temperature and working fluid pressure, the following assumptions can be made: assuming that one of the variables is in a constant state, study the relationship between the other two variables. There are the following three relational models: assuming that the working fluid flow is constant, establish the relationship model between the working fluid temperature and the working fluid pressure; assuming that the working fluid pressure is constant, establish the relationship model between the working fluid flow and the working fluid temperature; assuming that the temperature of the working fluid is constant, establish the relationship model between the working fluid flow rate and the working fluid pressure Among them, in the process of working fluid through the Roots power engine, the working fluid state of the intake part is recorded as state 1, and the working fluid state of the outlet part is recorded as state 2, which is represented by subscripts.

(1) When the working fluid flow is constant, the relationship between the working fluid temperature and the working fluid pressure.

Because the internal working process of Roots power machine is complex, in order to simplify, when the working medium does work in the cavity of Roots power machine, its internal flow process is treated as stable flow, that is, when the working medium flows through the internal space at any moment, all state parameters do not change with time^33^. At this time, the working fluid gas can be regarded as an ideal gas, which satisfies the formula 3.2:

3.2 $$PV = nRT$$

In the formula, *P* is the pressure of the working fluid, *V* is the volume of the gas, *n* is the amount of substance in the working fluid, *R* is the ideal gas constant, and *T* is the temperature of the working fluid.

Among them, the working fluid flow rate can be represented by the pipe cross-sectional area *S* and the working fluid flow velocity *v*, and the volume *V* can be represented by the flow rate *Q* and time *t*:

3.3 $$V = Qt$$

3.4 $$Q = Sv$$

After the Roots power machine performs work, the working fluid state of the intake and exhaust can be expressed as:

3.5 $$\frac{{QP_{1} t}}{{T_{1} }} = \frac{{QP_{2} t}}{{T_{2} }} + C_{1}$$

Among them, *C*_*1*_ is a constant related to the energy consumed by the Roots power machine. This relational model can be used as a similar model in the process of waste heat power generation.

(2) When the working fluid pressure is constant, the relationship between the working fluid flow and the working fluid temperature.

When the working fluid pressure is stable, this model can also be simplified to a steady flow state, and the working fluid gas at this time can be regarded as an ideal gas and treated with Eq. (3.2). After the Roots power machine has done work, the working fluid state of the intake and exhaust can be expressed as:

3.6 $$\frac{{Q_{1} Pt}}{{T_{1} }} = \frac{{Q_{2} Pt}}{{T_{2} }} + C_{2}$$

*C*_*2*_ is a constant related to the energy consumed by the Roots power machine. Similarly, this relationship model can be used as a similar relationship in the actual application process.

(3) When the working fluid temperature is constant, the relationship between the working fluid flow and the working fluid pressure.

When the temperature is constant, the relationship between gas pressure and flow rate can be expressed by Bernoulli Eq. ^34^:

3.7 $$P + \rho gh + \frac{1}{2}\rho v^{2} = C$$

The second term represents gravitational potential energy, the third term represents kinetic energy, and *C* is a constant. The application conditions of formula 3.7 include four aspects: in the flow system, the properties of the fluid do not change with time at any point; the Mach number of the fluid is less than 0.3, that is, the flow velocity does not exceed 102 m/s; the friction effect and the viscosity effect are negligible; the fluid unit Flowing along the streamlines, the streamlines do not intersect each other. Through the analysis and research on the waste heat recovery system, in general, the gas state in the waste heat recovery system meets the application conditions of Bernoulli equation. Since the gas will be buoyant in the air, the gravitational potential energy of the gas can be neglected, then the formula 3.7 can be expressed as:

3.8 $$P + \frac{1}{2}\rho v^{2} = C$$

After the Roots power machine performs work, the state change of the working fluid can be expressed as:

3.9 $$P_{1} + \frac{1}{2}\rho v_{1}^{2} = P_{2} + \frac{1}{2}\rho v_{2}^{2} + E$$

Among them, the relationship between flow rate and flow rate can be expressed by Eq. (3.4). *E* is a constant related to the energy consumed by the Roots power machine, and can be expressed as the energy consumed by the Roots power machine for a unit volume of gas.

After a large number of experiments in the early stage, it was found that the temperature change of the air inlet and outlet of the Roots power machine was not obvious, and the main influence on the speed was the working fluid pressure and the working fluid flow. In the waste heat recovery system, only the regulating valve is a controllable component. The opening and closing degree of the regulating valve directly affects the flow of the working fluid, so all variables must ultimately be related to the impact on the flow.

### Characteristics of flow control valve

The electric valve is responsible for the adjustment of the intake flow of the Roots waste heat power generation device. From the perspective of working mode, it is composed of an electronic controller and a mechanical actuator, including components such as a motor and a reducer. Because the electric actuator has a fast adjustment speed and can still maintain stable adjustment in a harsh factory environment, the application of electric control valves in industrial production is more common^35^.

Since the electric control valve is composed of two parts, an electric actuator and a valve body, its flow characteristics are also determined by the influence of these two aspects.

The valve body flow characteristic refers to the relationship between the relative flow of the medium flowing through the valve body and the relative opening of the valve body^36^. It can be expressed as:

3.10 $$\frac{Q}{{Q_{\max } }} = f(\frac{L}{{L_{\max } }})$$

The electric control valve selected in the Roots waste heat power generation device has a linear flow characteristic. So we can get:

3.11 $$\frac{{d\frac{Q}{{Q_{\max } }}}}{{d\frac{L}{{L_{\max } }}}} = k$$

After integration, the equation is obtained:

3.12 $$\frac{Q}{{Q_{\max } }} = k\frac{L}{{L_{\max } }} + c$$

when *L* = 0, *Q* = *Q*_*min*_, which means the minimum adjustable flow of the control valve; when *L* = *L*_*max*_, *Q* = *Q*_*max*_, which means the maximum adjustable flow of the control valve.

The actual flow rate through the regulating valve is not solely determined by the opening and structure of the valve, but is also restricted by the pressure difference between the front and rear of the valve body. In industrial field applications, the pressure drop in the intake pipe of the Roots waste heat power generation device will be distributed on the regulating valve and the pipes before and after it according to a certain rule. For the convenience of explanation, the ratio of the pressure drop concentrated on the valve and the total pressure drop in the pipeline when the valve is fully opened is taken as the s coefficient, which is used to describe the pressure drop distribution in the intake passage.

3.13 $$s = \frac{{\Delta P_{v} }}{{\Delta P_{v} + \sum_{i = 1}^{n} \Delta P_{i} }}$$

Among them, *ΔP*_*v*_ represents the pressure drop distributed on the valve; *ΔP*_*i*_ represents the pressure drop on the pipeline.

**Figure 5:**
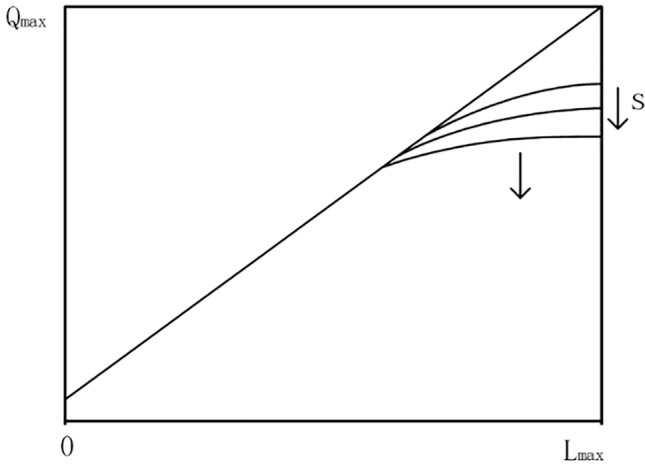
Flow characteristic curve of valve.

Figure 5 illustrates the flow characteristics of the linear control valve. If *s* = 1, it means that all the pressure in the pipeline is concentrated on the valve, and the pipeline resistance is zero, then the flow in the pipeline is completely determined by the valve, so the flow characteristic is a straight line in an ideal state. If s decreases, that is, the pipeline resistance increases, the pressure drop borne by the control valve decreases, which will cause the maximum adjustable flow rate of the valve to decrease. If *s* = 0, it means that all the pressure is concentrated on the pipeline, and the flow in the pipeline is completely determined by the resistance of the pipeline, and the valve can no longer adjust the flow.

Therefore, it can be determined that the inherent flow characteristics of the intake pipe regulating valve follow a certain law, but the adjustment effect on the flow will be distorted in actual work.

**Figure 6:**
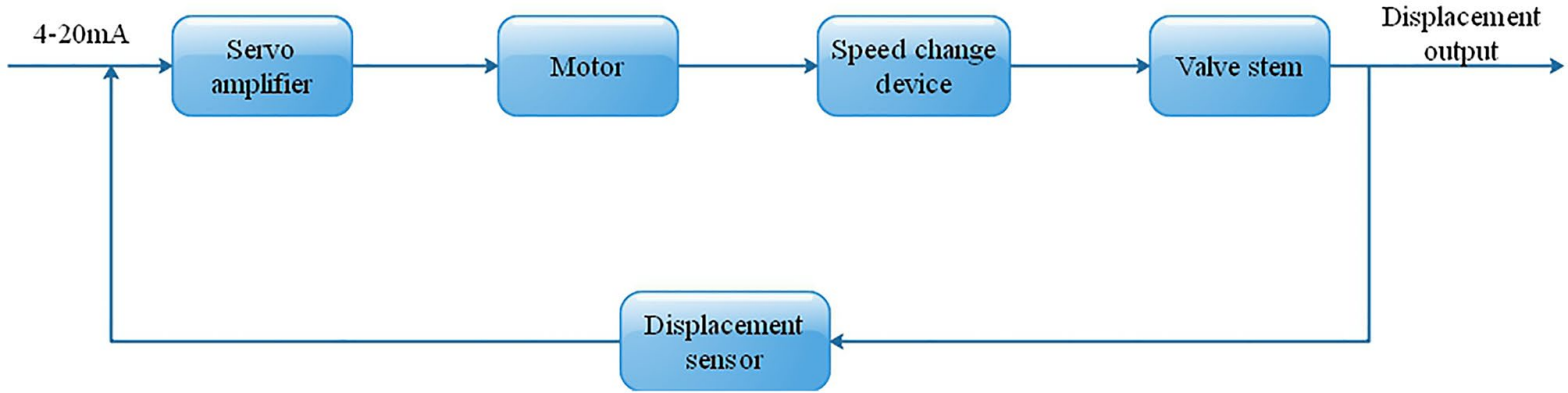
Block diagram of electric actuator.

The structure of the electric actuator mainly includes four parts: servo amplifier, motor, speed change device, valve stem and displacement sensor, as shown in Fig. 6. The servo amplifier amplifies the deviation between the standard current signal sent by the controller and the position signal of the actual displacement of the valve core as the drive signal of the motor, and sends it to the motor. The motor drives the speed change mechanism to produce linear displacement, and then drives the corresponding mechanical structure to change the valve opening and change the flow area.

In summary, it can be determined that the control valve is not a single output single output. Therefore, when the electric control valve is adjusted in flow, there is a problem of strong coupling of variables and non-linear fluctuations.

## Design of control system

### Analysis of traditional control effects

The ideal closed-loop control of the Roots waste heat power generation process is a single-input single-output closed-loop control of rotation speed. The adjustment goal of the Roots waste heat power generation process control system is to maintain the stability of the rotation speed, so the currently commonly used control method is the PID control method. The advantages of the PID control algorithm are mainly reflected in the ease of implementation. Through the control of the PID algorithm, the output is continuously adjusted and gradually tends to be close to and consistent with the input value.

In the design of the PID controller, the coefficient values of the three links of proportional, integral, and differential operations determine the performance of the controller, and the values need to be selected based on the characteristics of the controlled object. In addition to the adjustment of the three parameters having an impact on the control effect of the system, it is also necessary to point out that since the design basis of the PID controller is the process model obtained by the designer, the accuracy of the model will restrict the effect of the controller. In actual engineering control problems, the process model of the controlled object often does not completely match the nominal model obtained by the designer, which is often called model mismatch. In this case, the effect of the controller is restricted. Therefore, for the PID controller of the Roots waste heat power generation process, in this single-input single-output loop, the parameters affecting the output in the system have been fitted to the parameters under the standard operating conditions. This ignores the time-varying and non-linearity of other parameters in the actual Roots waste heat power generation process, and there are errors with actual working conditions.

After the above analysis, the reasons for the poor performance of the PID controller can be summarized:

1. Due to the simple algorithm, when the PID controller is dealing with the frequent and complicated Roots waste heat power generation device, the parameter tuning results are often not ideal.
2. The design of PID controller depends on the accuracy of the model of the controlled object. However, the coupling of parameters in the Roots waste heat power generation process is complicated and it is difficult to obtain an accurate model.
3. Due to the limitation of the design principle, it is difficult to simultaneously meet the requirements for the rapidity of tracking the set value and the stability of suppressing disturbances.

### Nonlinear multi-model adaptive closed-loop decoupling control

The waste heat recovery system has the characteristics of many variables, mutual correlation, multiple models and strong coupling, so it is necessary to introduce targeted control methods to solve these problems. For this reason, the research group introduced nonlinear multi-model adaptive closed-loop decoupling control into the Roots waste heat recovery device. This control method is mainly aimed at nonlinear multi-variable, non-minimum phase coupled systems, and is obtained by combining a one-step leading optimal weighted feedforward closed-loop decoupling strategy with a multi-model algorithm^37^.

For the waste heat recovery system, a non-linear multivariable system can be established:

4.1 $$A(z^{ - 1} )y(t + d) = B(z^{ - 1} )u(t) + v[X(t)]$$

In the formula, *A* and *B* are parameter matrices; ***u***(*t*) and *y*(*t*) are the input and output vectors of the system respectively; *X*(*t*) is the data vector composed of input and output sequences; *v* is the high-order nonlinearity of the system item.

For closed-loop decoupling design, the coupling between different channels in the system is regarded as measurable interference, and the feedforward method is used to eliminate it.The equations is obtained:

4.2 $$A(z^{ - 1} )y(t + d) = \overline{B} (z^{ - 1} )u(t) + \overline{{\overline{B} }} (z^{ - 1} )u(t) + v[X(t)]$$

4.3 $$\overline{B} (z^{ - 1} ) = diag[B_{ii} (z^{ - 1} )]$$

4.4 $$\overline{{\overline{B} }} (z^{ - 1} ) = B(z^{ - 1} ) - \overline{B} (z^{ - 1} )$$

Equation 4.3 is a diagonal polynomial matrix, which represents the relationship between input and output variables on the main channel. Equation 4.4 is a polynomial matrix with zero main diagonal elements, which represents the coupling relationship between different channels.

Introduce a weighted one-step leading optimal performance index:

4.5 $$J^{\prime}(t) = \frac{{\left\| {P(z^{ - 1} )y(t + d) - R(z^{ - 1} )w(t + d) + K(z^{ - 1} )v[X(t)]} \right\|}}{2} + \frac{{\left\| {Q^{\prime}(z^{ - 1} )u(t) + S^{\prime}(z^{ - 1} )u(t)} \right\|}}{2}$$

The weighted polynomial matrix is:

4.6 $$Q(z^{ - 1} ) = \frac{{[Q^{\prime}(0) + S^{\prime}(0)]Q^{\prime}(z^{ - 1} )}}{{F_{0} B_{0} + F_{0} C + K_{0} C}}$$

4.7 $$S(z^{ - 1} ) = \frac{{[Q^{\prime}(0) + S^{\prime}(0)]S^{\prime}(z^{ - 1} )}}{{F_{0} B_{0} + F_{0} C + K_{0} C}}$$

4.8 $$P(z^{ - 1} ) = F(z^{ - 1} )A(z^{ - 1} ) + z^{ - d} G(z^{ - 1} )$$

*K*_0_ is the regular matrix of *K*(*z*^-1^), F0 is the regular matrix of *F*(*z*^-1^), and *F*(*z*^-1^) is determined by Eq. 4.8, that is, *F*_0_ = *P*(0).

In order to ensure the existence of the current input ***u***(*t*), the weighted polynomial matrix should satisfy:

4.9 $$\det P(0) \ne 0,and\det [F_{0} B_{0} + Q(0) + S(0)] \ne 0$$

In order to make the closed-loop system stable, the weighted polynomial matrix should also satisfy:

4.10 $$\det \{ P(z^{ - 1} )B(z^{ - 1} ) + A(z^{ - 1} )[Q(z^{ - 1} ) + S(z^{ - 1} )]\} \ne 0,\left| z \right| \ge 1$$

At this time, the approximate dynamic decoupling of the closed-loop system of the Roots waste heat power generation device can be realized.

The recursive formula is used to directly identify the parameter matrix of the controller to generate control input. To this end, multiply both sides of Eq. 4.1 by *F*(*z*^-1^) and use Eq. 4.8 to obtain the controller parameter identification equation:

4.11 $$P(z^{ - 1} )y(t + d) = G(z^{ - 1} )y(t) + H(z^{ - 1} )u(t) + F(z^{ - 1} )v[X(t)]$$

That is, the optimal decoupling control law:

4.12 $$\varphi (t + d) = \Theta^{T} X(t) + \xi [X(t)]$$

The model of the controller parameter identification equation is defined as:

4.13 $$\hat{\varphi }_{1} (t + d) = \hat{\Theta }_{1} (t)^{T} X(t) + \xi [X(t)]$$

Use the following identification methods to calibrate online:

4.14 $$\hat{\Theta }_{1} (t) = {\text{proj}}\{ \hat{\Theta }^{\prime}_{1} (t)\}$$

4.15 $$\hat{\Theta }^{\prime}_{1} (t) = \hat{\Theta }^{\prime}_{1} (t - d) + \frac{{a_{1} (t)X(t - d)e_{1} (t)^{T} }}{{1 + X(t - d)^{T} X(t - d)}}$$

4.16 $$if:\left\| {e_{1} (t)} \right\| > 2M,a_{1} (t) = 1;else:a_{1} (t) = 0$$

4.17 $$e_{1} (t) = \varphi (t) - \hat{\varphi }_{1} (t)$$

Proj is a projection operator.

According to the optimal control law and the principle of deterministic equivalence, the adaptive closed-loop decoupling controller based on the linear model can be obtained:

4.18 $$\hat{\Theta }_{1} (t)^{T} X(t) = R(z^{ - 1} )w(t + d) - [Q(z^{ - 1} ) + S(z^{ - 1} )]u(t)$$

Adaptive closed-loop decoupling controller based on nonlinear model:

4.19 $$\hat{\Theta }_{2} (t)^{T} X(t) + \hat{\xi }[X(t)] = R(z^{ - 1} )w(t + d) - [Q(z^{ - 1} ) + S(z^{ - 1} )]u(t) - \hat{\eta }[X(t)]$$

4.20 $$\hat{\eta }[X(t)] = [\frac{{Q(z^{ - 1} )}}{{\hat{H}_{2} (t,z^{ - 1} )}}]\hat{\xi }[X(t)]$$

Selection of switching function criteria:

4.21 $$J_{i} (t) = \sum\limits_{l = d}^{t} {\frac{{a_{j} (l)[\left\| {e_{j} (l)} \right\|^{2} - 4M^{2} ]}}{{2[1 + X(l - d)^{T} X(l - d)]}}} + c\sum\limits_{l = t - N + 1}^{t} {[1 - } a_{j} (l)]\left\| {e_{j} (l)} \right\|^{2}$$

4.22 $$if:\left\| {e_{j} (t)} \right\| > 2M,a_{j} (t) = 1;else:a_{j} (t) = 0$$

Among them, *N* is a positive integer; *c* is a predetermined constant greater than or equal to 0; *j* = 1,2, *j* = 1 means linearity, and *j* = 2 means nonlinearity.For each time *t*, compare *J*_1_(*t*) and *J*_2_(*t*), find the smallest J_*_(*t*), and select the direct adaptive decoupling controller u_*_(*t*) corresponding to J_*_(*t*).

## Simulation and experiment

In industrial production, PID control is the most commonly used nonlinear control method. Before, the preliminary research results of the research group is based on PID control roots waste heat power generation system. Therefore, the PID control method is suitable as the control method. This part will verify the control effect of nonlinear multi-model adaptive decoupling control from two aspects of simulation and practical application.

### Simulation analysis

**Figure 7:**
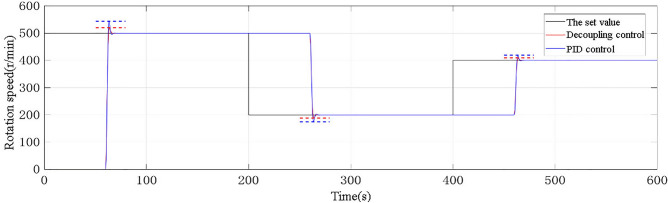
Curve of tracking performance.

The PID controller used in the Roots waste heat power generation process is calculated through the Ziegler-Nichols PID parameter tuning table. The test method is to take the step signal as the input signal of the system, and the initial signal amplitude is 500 r/min. When *t* = 200 s, the step signal -300 is added. When *t* = 400 s, the step signal is + 200, so that the input of the model corresponds to the speed setting values of 500, 200, and 400 r/min. Finally, the tracking performance curve of the system is shown in Fig. 7. The dotted line in the figure indicates the peak value.

In the figure, the black curve represents the speed set value, while the red curve represents the speed under decoupling control and the blue curve represents the speed under PID control. It is obvious from the figure that the overshoot of decoupling control is smaller than that of PID control. Through analysis and calculation of the data in the figure, Table 2 can be obtained. It can be seen that: under the condition that the controlled object model is accurate, the adjustment speed of the two controllers is similar. PID controller is designed based on the nominal model, so it performs well in the control of the nominal model, but its overshoot is nearly two times that of the decoupled control. According to the data obtained from the simulation curve, the IATE ratio of the system output under the action of decoupling control and PID controller is calculated to be 0.67. In general, the decoupling control method is superior to the conventional PID controller both in overshoot and regulation time.

**Table 2:** Tracking performance.

Performance	Decoupling control	PID control
Overshoot	3.9%	8%
Adjustment time	66s	70s

### Experimental test

In order to verify whether the nonlinear multi-model adaptive closed-loop decoupling control can achieve the effect required by the control index under actual working conditions, an experimental platform needs to be built to verify the control method. The purpose of the experiment is to determine whether the controller can keep the speed within the range of ± 7% of the set speed when the air source is disturbed.

**Figure 8:**
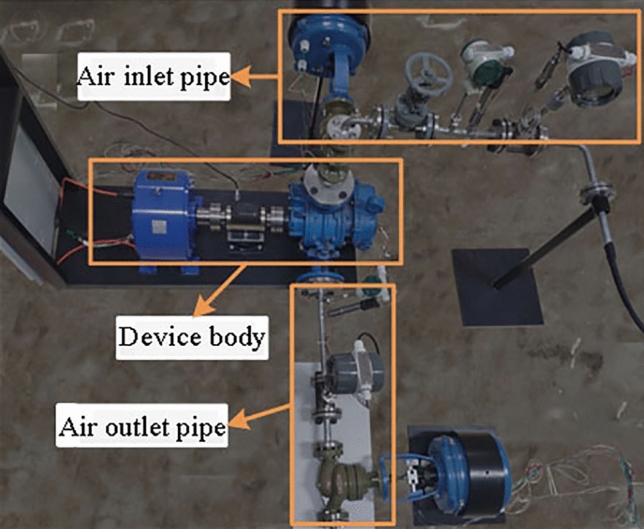
The overall structure of the experimental platform.

The structure of the experimental platform is similar to Fig. 3. Under laboratory conditions, the experimental platform as shown in Fig. 8 is built according to the structure of the waste heat recovery system. The experimental platform includes an air inlet pipe, a device body and an air outlet pipe. Low quality waste heat enters through the inlet pipe, which is installed with thermometer, pressure gauge, flow meter, electric control valve and cut-off valve. The main body of the device is used for low quality waste heat to do work and output mechanical energy. The air outlet pipeline is used to discharge low quality waste heat after work, and the air outlet pipeline is also installed with relevant sensors for monitoring.

**Figure 9:**
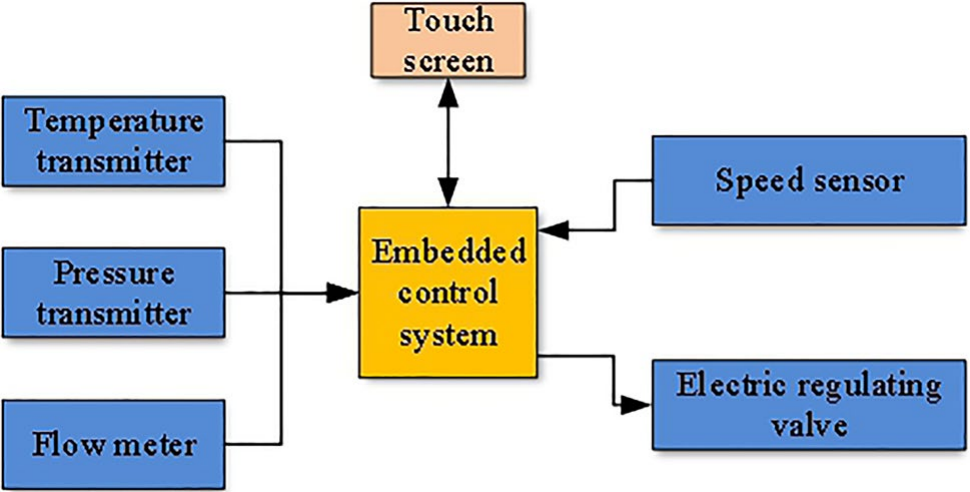
The connection of the control system.

The specific connection of the control system is shown in Fig. 9.

**Figure 10:**
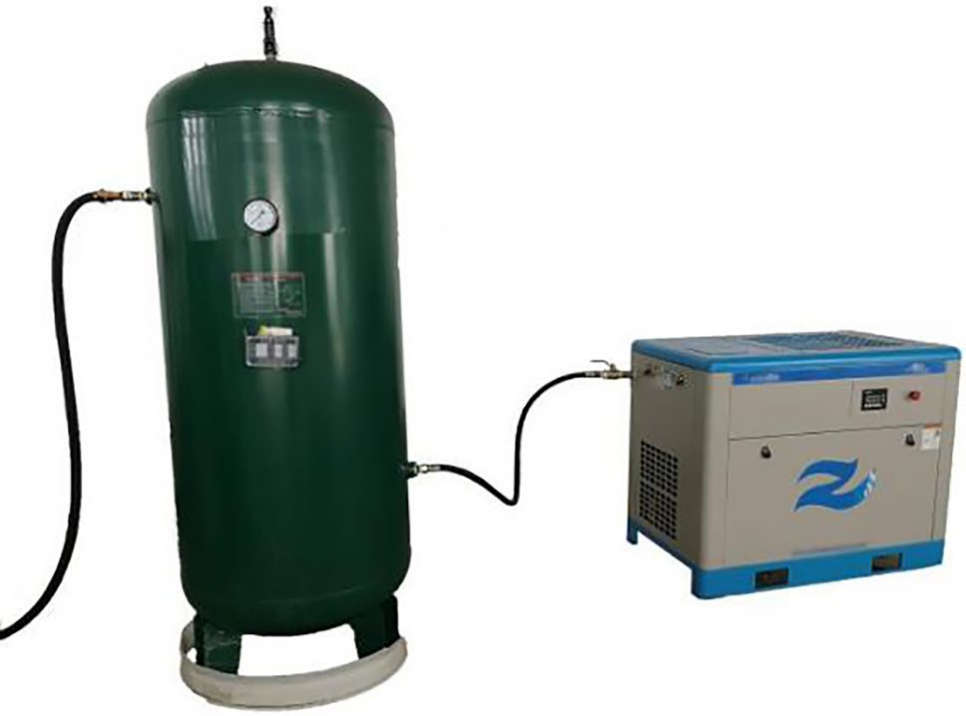
Gas source.

In this experiment, a gas storage tank with low-quality waste heat steam was used as the gas source. As shown in Fig. 10. The compressed air stored in the air storage tank is up to 1.2 MPa, which meets the experimental requirements of low quality waste heat. Adopt no-load experiment method to detect the output rotation speed.

Anti-interference ability is the most intuitive embodiment of the stability of the control system. The actual operating condition is different from the simulation condition. The simulation condition is more ideal, but the actual operating condition is easily disturbed by the external environment. In order to verify the controller's ability to suppress interference, when the test device is in a stable working condition, the opening of the outlet valve of the Roots power machine is continuously adjusted, and the parameters of other components remain unchanged, so that the airflow continues to fluctuate. After the experiment, the system was updated and the same experiment was carried out with PID control method as the experimental control.

The specific experimental steps are as follows:

1. Proofread the control system circuit schematic diagram and wiring diagram, and comprehensively check the circuit wiring according to the relevant drawings, to ensure the reliability of the control system hardware;
2. Power on to test whether the controller, each sensor and the actuator are in normal working condition;
3. Write the control test program into the controller;
4. Close the main valve, open the air compressor, set the output gas pressure of the gas storage tank to 0.6mpa, which is the rated input pressure;
5. Slowly open the main valve, adjust the intake valve to the preset position, roots power machine operation;
6. Adjust the main valve to keep the rotating speed of roots power machine at 600 r/min;
7. After the stable operation of the equipment, manually fine-tune the main valve to simulate the state of interference and record data at the same time^38^.

**Figure 11:**
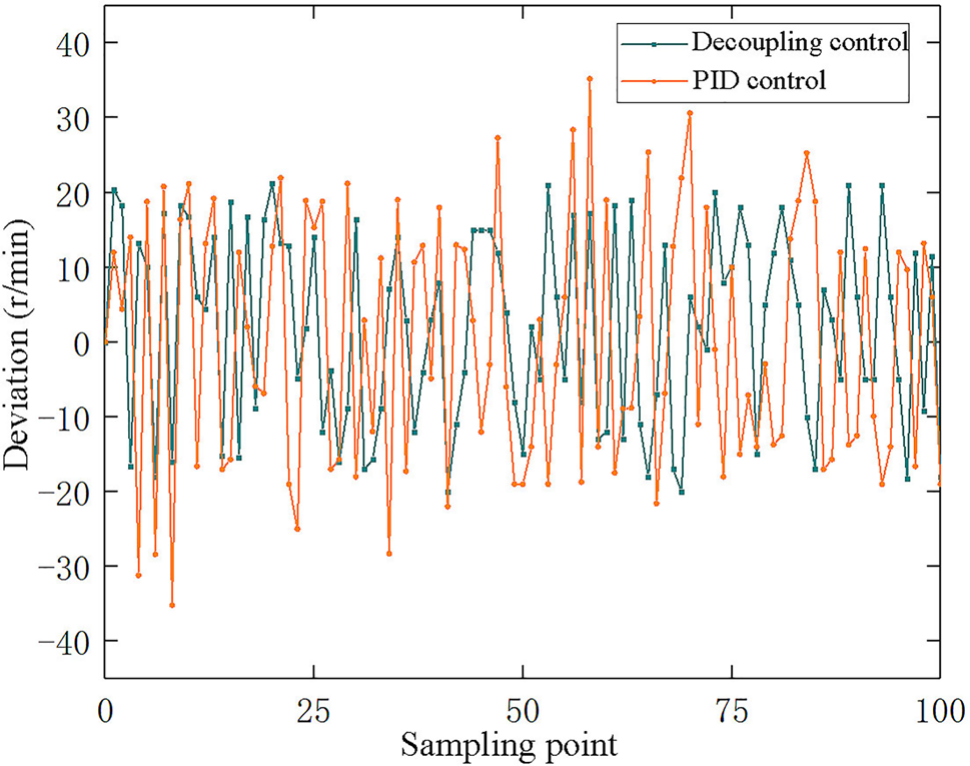
Speed deviation contrast curve.

Collect the output rotation speed information at this time and use it to simulate the change of the device operating condition when it is disturbed by the outside world. The obtained rotation speed deviation curve is shown in Fig. 11.

In the figure, green represents the decoupling control curve and yellow represents the PID control curve. It can be seen that the upper and lower boundary of PID control curve is larger than that of decoupling control, that is, the deviation under PID control is larger. This shows that the decoupling control has stronger inhibition ability than the conventional PID controller when the system is disturbed. The signal processing mode of PID controller is much simpler than decoupling control. PID control directly takes the difference between the reference given and the output feedback as the control signal, resulting in the contradiction between response speed and overtone. Decoupling control is based on parameters and model to calculate the size of the adjustment, the middle through a complex function operation process. So decoupling control makes the system more stable in the face of external parameter disturbance.

The test of the tracking performance of the set value reflects the dynamic adjustment performance of the controller as well as the robust tracking performance. When the system runs stably near the operating point of 500 r/min speed, the set value of roots power machine speed is raised to 600 r/min. Obtain the data shown in Table 3.

**Table 3:** Track performance data.

Serial number	Desired speed (r/min)	Decoupling control Actual speed (r/min)	PID control Actual speed (r/min)
1	600	585.5	572.3
2	600	598.9	579.9
3	600	613.5	583.1
4	600	617.8	599.7
5	600	620.3	625.2
…	…	…	…
46	600	606.3	599.8
47	600	605.5	614.8
48	600	594.8	602.8
49	600	597.3	599.8
50	600	599.6	578.8

Non-linear multi-model adaptive closed-loop decoupling control performs faster than conventional PID controllers in dynamic performance, and has a smaller overshoot. The speed deviation in the experiment is not higher than 21.4 r/min, which is within the allowable fluctuation range. The adjustment time for tracking the set value does not exceed 65.3 s. After the speed is stable, when the sensor detects the change of air source, the controller calculates how to adjust the regulating valve according to the change of parameters and the coupling model of the system. In the adjustment process, the controller continuously modifies the regulating valve according to the closed-loop feedback signal, and finally realizes the speed stability. The experimental results show that when the air source changes, the controller can respond quickly to change the rotation speed of roots motor, and realize the tracking of the set value.

The actual working condition is often not consistent with the simulation results, and there is often uncertainty. The uncertainty in this system is mainly divided into two kinds: model mismatch and small fluctuation disturbance of air source. Model mismatch refers to the fact that the process model of the controlled object often does not match the nominal model obtained by the designer in the actual engineering control problem. In this experiment, the approximate coupling model of the waste heat utilization system has been given in chapter 3. As the approximate model is included in the models, the model mismatch exists in the experimental process, which will also affect the stability of the control system. The experimental results show that the control system can keep the speed stable within the control index in the face of model mismatch, which further proves that the nonlinear multi-model adaptive closed-loop decoupling control has strong anti-interference ability. Small fluctuation interference refers to the unstable fluctuation of gas source, namely the main interference source of this experiment. The uncertainty of small fluctuation interference is reflected in the uncertainty of fluctuation amplitude and fluctuation frequency. Experimental data also prove that nonlinear multi-model adaptive closed-loop decoupling control has better control effect than PID control in the face of small wave disturbance.

## Conclusion

The research group designed a Roots-type waste heat recovery device for low-quality waste heat recovery. However, it is difficult for this device to keep the rotation speed stable when the air source fluctuates. Because the system has the characteristics of large lag, multi-variable and strong coupling, in order to solve this problem, the research group carried out further research on the device. The specific research content includes the following aspects:

1. By analyzing the model of low-quality waste heat recovery system, it is found that there are many variables in the system with strong coupling.
2. Through the analysis of input variables and output variables, variable coupling models under different conditions are obtained. And the parameter index of fluctuation range deviation is determined to be ± 7%.
3. Traditional PID control has the characteristics of hysteresis, slow response speed and poor stability. Aiming at the defects of traditional PID, the research group designed nonlinear multi-model adaptive closed-loop decoupling control. The control method can be used to control the rotational speed under the condition of air source fluctuation based on variable coupling model.
4. Complete the design of decoupling controller based on the parameters of waste heat recovery system. The simulation results show that the adjusting time of decoupling control is shortened by about 4 s. In terms of overshoot, the maximum overshoot of decoupling control is 3.9%, about half of that of conventional PID control, which has obvious advantages.
5. In the experimental process, the decoupling control has stronger inhibition ability and better stability than the conventional PID controller. In addition, when the air source changes, the controller can respond quickly to realize the tracking of the set value, and the deviation after stability is not more than ± 7%, to meet the control index. Therefore, when the device is applied to the actual industrial field, the nonlinear multi-model adaptive closed-loop decoupling control has certain availability.
